# Hepatoprotective activities of a sesquiterpene-rich fraction from the aerial part of *Cichorium glandulosum*

**DOI:** 10.1186/1749-8546-7-21

**Published:** 2012-09-29

**Authors:** Wei-Jun Yang, Yu-Qin Luo, Haji Akber Aisa, Xue-Lei Xin, Z Totahon, Yan Mao, Meng-Ying Hu, Lei Xu, Rui-Ping Zhang

**Affiliations:** 1 Xinjiang Key Laboratory of Plant Resources and Natural Products Chemistry, Xinjiang Technical Institute of Physics and Chemistry, Chinese Academy of Sciences, Urumqi, 830011, China; 2 Graduate University of the Chinese Academy of Sciences, Beijing, 100049, China; 3 Xinjiang Key Laboratory of Xinjiang Uygur Medicine, Xinjiang Institute of Materia Medica, Urumqi, 830004, China

## Abstract

**Background:**

*Cichorium glandulosum* Boiss. et Huet is used for treatment of liver disorders, and its effects are attributed to sesquiterpenes. This study aims to investigate the hepatoprotective effects of a sesquiterpene-rich fraction (SRF) from the aerial part of *C. glandulosum* on carbon tetrachloride (CCl_4_)-induced acute hepatotoxicity in mice, and on priming with Bacillus Calmette–Guerin (BCG) followed by lipopolysaccharide (LPS)-induced immunological liver injury in mice.

**Methods:**

SRF was suspended in water and administered to mice at 0.05, 0.10 and 0.20 g/kg body weight for 7 consecutive days. An active control drug (bifendate pills) was suspended in distilled water and administered to mice at 0.40 g/kg body weight for 7 consecutive days. Hepatotoxicity was induced by intraperitoneal injection of 0.1% CCl_4_ (0.2 mL/mouse) at 13 h before the last drug administration, or by tail intravenous injection of BCG (0.2 mL/mouse) before the first drug administration and LPS (0.2 mL/mouse; 8 μg) at 15 h before the last drug administration. Blood samples and the livers were collected for evaluation of the biochemical parameters of aspartate aminotransferase (AST), alanine aminotransferase (ALT) and total bilirubin (TBIL).

**Results:**

SRF significantly reduced the impact of CCl_4_ toxicity. The highest dose of SRF (0.20 g/kg) was the most effective, reflected by significant reductions in the levels of AST (*P* = 0.001), ALT (*P* = 0.000) and TBIL (*P* = 0.009). The serum enzymatic levels induced by BCG and subsequent LPS injection were significantly and dose-dependently restored by SRF, reflected by significant reductions in the levels of AST (*P* = 0.003), ALT (*P* = 0.003) and TBIL (*P* = 0.007) for the highest dose of SRF (0.20 g/kg).

**Conclusion:**

SRF is hepatoprotective in animal models of chemical and immunological acute liver injury.

## Background

The liver is a vital organ that is vulnerable to many diseases, such as hepatitis A, B, C and E, alcohol damage, fatty liver, cirrhosis, cancer and drug damage [1,2]. Free radicals are the main causes of liver diseases, and liver ailments remain a serious health problem [3]. Carbon tetrachloride (CCl_4_) is frequently used as a chemical inducer of experimental tissue damage [4-6], owing to its production of a free radical, trichloromethyl radical (·CCl_3_). Bacillus Calmette–Guerin (BCG) and subsequent lipopolysaccharide (LPS) injection provokes hepatic injury in mice [7], and is considered to be a useful experimental model for immunological liver injury [8].

Despite studies for a decade as well as advancement of our understanding of the molecular pathogenesis of liver diseases, the effective therapeutic interventions for liver diseases are still limited [5]. Antioxidant therapy inhibits deleterious oxidative changes, and has always been considered to be an important tool for liver disease treatments. Medicinal plants, especially those with traditional use, are considered to be a rich source of new effective drugs.

*Cichorium glandulosum* Boiss. et Huet was reported effective as a cholagogic and diuretic agent to improve the appetite, to increase digestion, and to cure various types of liver diseases, *etc.*[9]. The effects of dried roots, seeds, and aerial part of *C. glandulosum* have been well documented during long-term clinical practice [10]. The pharmacologically active constituents of *C. glandulosum* include a number of sesquiterpenoids and flavonoids, such as lactucin, lactucopicrin, 11*β*,13-dihydrolactucin and esculetin from the roots of *C. glandulosum*[11,12], and cichoriin quercetin-3-*O-β-D*-glucuronide and kaempferol-3-*O-β-D*-glucuronide from the aerial part of *C. glandulosum*[13]. These compounds demonstrate significant anticancer [14,15], antimalarial [16], analgesic and sedative [17] and anti-inflammatory [18] activities.

This study aims to investigate the *in vivo* activity of SRF against experimental liver injury caused by administration of CCl_4_ and BCG + LPS, respectively.

## Methods

### Chemicals and reagents

CCl_4_ was purchased from Tianjin No. 3 Chemical Reagent Factory (China) (batch no. 20110925). Ybarra extra virgin olive oil was produced by Aceites Ybarra S.A. (Spain) (batch no. 20101026). BCG vaccine for intradermal injection was produced by Shanghai Institute of Biological Products Co. Ltd. (China) (batch no. 201011054–1). Sterilized water for injection was produced by Jiangsu Tianhe Disainuo Pharmaceutical Co. Ltd. (China) (batch no. 20110523.2). LPS was purchased from Sigma Corporation (USA) (batch no. L-2880). Sodium chloride (0.9%) injection was produced by Sinopharm Group Xinjiang Pharmaceutical Co. Ltd. (China) (batch no. 20120210). Assay kits for serum aspartate aminotransferase (AST), alanine aminotransferase (ALT) and total bilirubin (TBIL) were purchased from Biosino Bio-Technology & Science Inc. (China) (batch no. 110861, 111761 and 110721 respectively). All other chemicals were of analytical grade.

### Plant materials

*C. glandulosum* Boiss. et Huet was collected in Hutan County, China, in November 2007. The plant was identified by Dr. LY Zhang (Xinjiang Institute of Ecology and Geography, Chinese Academy of Sciences, Urumqi, China), where voucher specimens (no. 051054 and 051055) have been deposited.

### Preparation of SRF

The air-dried aerial part of *C. glandulosum* (125 kg) was extracted twice with ethanol (40%) at 60°C for 3 h with refluxing in a water bath at a raw material/extractant ratio of 1:15. The ethanol was vacuum-distilled from the combined extract using a rotary vacuum evaporator (Rotavapor R-220; Buchi, Switzerland) at 60°C until a relative density of 1.01–1.05 (60°C) was achieved with the density bottle method [19]. The aqueous residue was then added (1150 mL/min) to a column packed with HPD-100 macroporous adsorption resin (Cangzhou Bonchem Co., Ltd., China) at an aqueous residue/resin ratio of 6:1 (v/v) and incubated for 6 h to allow absorption. Subsequently, the resin was washed with water at 4× the resin volume (1960 mL/min), and the eluent was discarded. The resin was then washed with ethanol (60%) at 6× the resin volume (1150 mL/min). The eluent was concentrated using the rotary vacuum evaporator and vacuum-dried to obtain SRF (950 g).

### Animals

Kunming mice (18–22 g) of either sex were purchased from the Experimental Animal Center of the Center for Disease Control of Xinjiang (China). The mice were kept in a specific room at a temperature at 21–23°C on a 12-h/12-h light/dark cycle (lights on from 08:00 h to 20:00 h), and provided with rodent chow and water *ad libitum*. The investigation conformed to the Guide for the Care and Use of Laboratory Animals (NRC), 2010.

### CCl_4_-induced hepatotoxicity

The protective effect of SRF treatment against CCl_4_-induced hepatotoxicity in mice was evaluated in a 7-day study. The animals were arbitrarily divided into six experimental groups with 10 mice/group. Group I served as a control and received distilled water only (0.2 mL/10 g body weight) during the experiment. Group II was given distilled water (0.2 mL/10 g body weight) for 7 days before CCl_4_ intoxication and served as a hepatotoxicity control group. Group III was given bifendate (0.40 g/kg body weight) for 7 days before CCl_4_ intoxication and served as a positive control group. Groups IV, V and VI were prophylactically treated perorally for 7 days with three different doses of SRF suspension (0.05, 0.10 and 0.20 g/kg/day, respectively). The mice in groups II–VI received an intraperitoneal injection of CCl_4_ (0.2 mL/mouse of 0.1% CCl_4_ solution in olive oil) 13 h before the final administration. The control group was intraperitoneally treated with an equal amount of olive oil. The animals were euthanized at 1 h after the CCl_4_ intoxication and olive oil treatment. Blood samples were collected for evaluation of the biochemical parameters.

### BCG + LPS-induced hepatotoxicity

To examine the effect of SRF on BCG + LPS-induced liver injury, mice were arbitrarily divided into six experimental groups with 10 mice/group. Group I was a control group, group II was a hepatotoxicity control group, group III was a positive control group and groups IV, V and VI were SRF pretreatment groups. Each group was given a tail intravenous injection of BCG (0.2 mL/mouse) before the first drug administration. Groups IV–VI were orally administered different doses of SRF (0.05, 0.10 and 0.20 g/kg body weight, respectively) once a day for 7 days. Group I was given distilled water only (0.2 mL/10 g body weight) and group III was orally administered bifendate (0.40 g/kg body weight). On day 7, at 15 h before the last administration, groups II–VI were given a tail intravenous injection of LPS in normal saline (0.2 mL/mouse; 8 μg) and group I was given a tail intravenous injection of an equal amount of saline solution. Blood samples for the biochemical analyses were taken at 1 h after the last administration.

### Determination of biochemical markers of hepatic injury

The collected blood samples were centrifuged (877 × *g*, 10 min, 4°C) and the serum samples were separated and stored at −20°C until analysis. The activities of AST, ALT and TBIL were determined using Aspartate Aminotransferase Kit, Alanine Aminotransferase Kit and Total Bilirubin Kit.(Bilsino Biotechnology Company Ltd., China). Enzyme activities were expressed as international units (U/l or μmol/L).

### Histological examination

Mice were euthanized under light ether anaesthesia at 1 h after the last dosage and the livers were removed and washed with normal saline. The liver tissues were fixed in 10% formalin, dehydrated in a series of ethanol solutions and embedded in paraffin. The paraffin-embedded tissues were cut into 5–6-μm sections,and stained with haematoxylin and eosin (HE). The histopathological characteristics were observed and recorded with an HPLAS-1000 Colorized Pathology Image Analyzer (Tongji Medical University Qian-ping Image Engineering Company, China).

### Statistical analysis

Results were expressed as the mean ± standard deviation (SD). Statistical analyses of data were performed with the SPSS 16.0 statistical package (IBM, USA). All statistical comparisons were performed by one-way ANOVA followed by Tukey’s test. *P* values of less than 0.05 were considered statistically significant and *P* values of less than 0.01 were considered highly significant. The dose-dependent relationships were visually determined.

## Results

### Effects of SRF on CCl_4_-induced liver injury

The effects of SRF on the biochemical markers in mice with CCl_4_-induced liver injury are shown in Table 1. After a single injection of CCl_4_, the activities of AST (*P* = 0.000) and ALT (*P* = 0.000) in group II were significantly increased to 968.58 ± 439.52 and 984.98 ± 381.14 U/l, respectively, and TBIL was elevated to 10.09 ± 2.37 μmol/L, compared with group I (213.76 ± 33.81 U/l, 62.87 ± 10.84 U/l and 9.49 ± 2.21 μmol/L, respectively). The activities of AST (*P* = 0.000), ALT (*P* = 0.004) and TBIL (*P* = 0.010) were significantly reduced in group III administered bifendate -(0.40 g/kg body weight). Pretreatment with different doses of SRF in groups IV, V and VI (0.05, 0.10 and 0.20 g/kg body weight, respectively) for 7 days reduced the activities of AST, ALT and TBIL compared with the group II- Of these, the highest dose of SRF (0.20 g/kg body weight) was the most effective, reflected by significant reductions in the levels of AST (*P* = 0.001), ALT (*P* = 0.000) and TBIL (*P* = 0.009). The effects were dose-dependent, but the large SDs in all three SRF-pretreated groups indicated persistence of inter-individual variability in drug responses between the mice-, taking into account the large number of animals in each group (Table 1).

**Table 1:** **Protective effects of SRFon CCl**_**4**_**-induced increases in AST,ALT and TBIL**

**Groups**	**Pretreatment (g/kg)**	**AST (U/l)**	**ALT (U/l)**	**TBIL (μmol/L)**
Group I	Vehicle	213.76 ± 33.81	62.87 ± 10.84	9.49 ± 2.21
Group II	Vehicle	968.58 ± 439.52^**^	984.98 ± 381.14^**^	10.09 ± 2.37
Group III	0.40	426.11 ± 145.43^##^	133.47 ± 71.92^##^	7.13 ± 2.24^##^
Group IV	0.05	779.79 ± 401.47^#^	714.15 ± 317.77^#^	7.90 ± 3.04
Group V	0.10	676.55 ± 382.08^#^	681.09 ± 261.49^#^	7.69 ± 1.14^#^
Group VI	0.20	380.09 ± 179.40^##^	279.35 ± 164.26^##^	7.44 ± 0.95^#^

Values are expressed as the mean ± SD of 10 mice/group.

**P < 0.01, significant difference *vs.* the group I.

^#^*P* < 0.05, significant difference *vs.* the group II.

^##^*P* < 0.01, significant difference *vs.* the group II.

**Figure 1:**
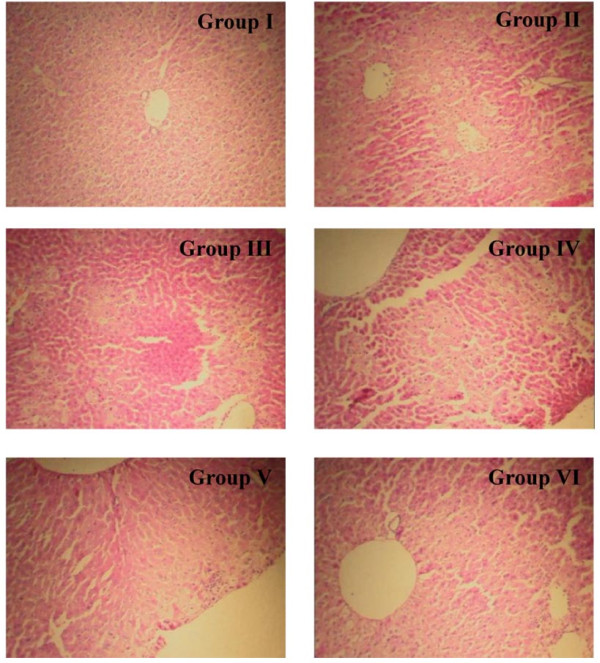
**Histopathologic sections of theliver in the CCl**_**4**_**-induced model.** (**Group I**) Liver sections of normal healthy mice show normal arrangements of cells in the liver lobules. (**Group II**) Hepatocyte necrosis and evident vacuolation of hepatocytes are observed in liver sections of CCl_4_-treated mice. (**Group III** and **Group IV**) Liver sections of mice pretreated with bifendate at 0.40 g/kg body weight (**Group III**) or SRF at 0.05 g/kg body weight (**Group IV**) show mild vacuolation of hepatocytes. (**Group V**) Mice pretreated with SRF (0.10 mg/kg body weight) show very mild hepatocyte necrosis and mild vacuolation of hepatocytes. (**Group VI**) Liver sections of mice pretreated with SRF (0.20 mg/kg body weight) show marked improvement in the histology compared with the CCl_4_-treated control group. HE staining; 100× magnification.

The histological observations supported the results of the serum enzyme assays. The liver sections in group I showed the normal lobular architecture and normal hepatic cells with a well-preserved cytoplasm and well-defined nucleus and nucleoli (Figure 1). Histopathological examination of the liver sections in group II showed centrilobular necrosis, ballooning degeneration, inflammatory infiltration and fatty changes. The liver sections in groups IV, V and VI revealed that SRF was able to prevent the development of histopathological changes in a dose-dependent manner. The liver sections of mice pretreated with the highest dose (0.2 g/kg body weight) showed a well-preserved architecture (Figure 1, Table 2).

**Table 2:** **Effects of SRF onhistopathological findings in micewith CCl**_**4**_**-induced liver damage**

**Groups**	**AHN**		**AHFC**		**AII**	
	**+**	**++**	**+**	**++**	**+**	**++**
Group I	0	0	2	0	2	0
Group II	4	1	3	1	3	0
Group III	2	1	2	0	2	0
Group IV	2	2	3	0	3	0
Group V	1	1	4	0	2	0
Group VI	2	0	4	0	1	0

A total of 10 mice were examined per group.

AHN, animals with hepatocellular necrosis; AHFC, animals with hepatocellular fatty change; AII, animals with inflammatory cell infiltration.

### Effects of SRF on BCG + LPS-induced liver injury

The effects of SRF on the biochemical markers in mice with BCG + LPS-induced liver injury are shown in Table 2. After a single injection of BCG + LPS, the activities of AST (*P* = 0.021) and ALT (*P* = 0.009) in group II were significantly increased to 222.44 ± 25.93 and 60.17 ± 7.43 U/l, respectively, and TBIL was elevated to 7.58 ± 1.41 μmol/L, compared with group I (197.02 ± 18.24 U/l, 51.67 ± 5.64 U/l, and 7.34 ± 1.41 μmol/L, respectively). The activity of ALT was significantly decreased (*P* = 0.000) with reductions in AST and TBIL in group III administered bifendate -(0.40 g/kg body weight). Pretreatment with different doses of SRF in groups IV, V and VI (0.05, 0.10 and 0.20 g/kg body weight, respectively) for 7 days reduced the activities of serum AST, ALT and TBIL compared with group II. The effects were dose-dependent, but the large SDs in all three pretreatment groups indicated the persistence of inter-individual variability in the drug responses between the mice, taking into account the large number of animals in each group (Table 3).

**Table 3:** **Protective effects of SRFon BCG + LPS-inducedincreases in AST, ALTand TBIL**

**Groups**	**Pretreatment (g/kg)**	**AST (U/l)**	**ALT (U/l)**	**TBIL (μmol/L)**
Group I	Vehicle	197.02 ± 18.24	51.67 ± 5.64	7.34 ± 1.41
Group II	Vehicle	222.44 ± 25.93^*^	60.17 ± 7.43^**^	7.58 ± 1.41
Group III	0.40	206.72 ± 31.70	37.04 ± 6.54^##^	7.42 ± 1.18
Group IV	0.05	220.04 ± 22.31	61.50 ± 7.94	6.80 ± 1.37
Group V	0.10	208.13 ± 16.99	56.37 ± 7.56	6.63 ± 0.77
Group VI	0.20	186.14 ± 20.44^##^	48.66 ± 7.27^##^	6.15 ± 0.46^##^

Values are expressed as the mean ± SD of 10 mice/group.

*P < 0.05, significant difference *vs.* the group I.

**P < 0.01, significant difference *vs.* the group I.

^#^*P* < 0.05, significant difference *vs.* the group II.

^##^*P* < 0.01, significant difference *vs.* the group II.

**Figure 2:**
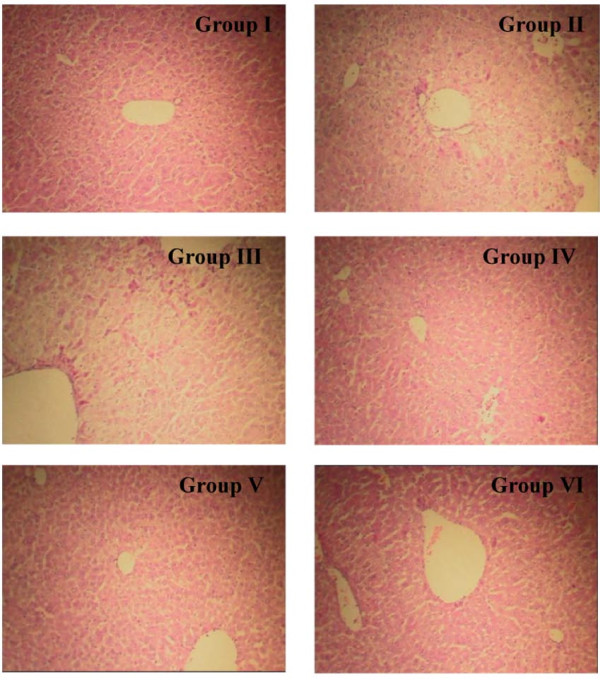
**Histopathologic sections of theliver in the BCG+ LPS-induced model.** (**Group I**) Liver sections of normal healthy mice show normal arrangements of cells in the liver lobules. (**Group II**) Hepatocyte necrosis and evident vacuolation of hepatocytes are observed in liver sections of BCG + LPS-treated mice. (**Group III** and **Group IV**) Liver sections of mice pretreated with bifendate at 0.40 g/kg body weight (**Group III**) or SRF at 0.05 g/kg body weight (**Group IV**) show mild vacuolation of hepatocytes. (**Group V** and **Group VI**) Mice pretreated with SRF at 0.10 mg/kg body weight (**Group V**) or 0.20 mg/kg body weight (**Group VI**) show marked improvement in the histology compared with the CCl_4_-treated control group. HE staining; 100× magnification.

The histological observations supported the results of the serum enzyme assays. The liver sections in group I showed the normal lobular architecture and normal hepatic cells with a well-preserved cytoplasm and well-defined nucleus and nucleoli. Histopathological examination of the liver sections in group II showed centrilobular necrosis, ballooning degeneration, inflammatory infiltration and fatty changes. The liver sections in groups IV, V and VI revealed that SRF was able to prevent the development of histopathological changes in a dose-dependent manner. The liver sections of mice pretreated with the highest dose (0.2 g/kg body weight) showed a well-preserved architecture (Figure 2, Table 4).

**Table 4:** **Effects of SRF onhistopathological findings in micewith BCG + LPS-inducedliver damage**

**Groups**	**AHN**		**AHFC**		**AII**	
	**+**	**++**	**+**	**++**	**+**	**++**
Group I	0	0	2	0	0	0
Group II	5	1	7	0	5	1
Group III	3	1	7	0	3	1
Group IV	2	0	6	0	2	0
Group V	1	0	7	0	1	0
Group VI	1	0	5	0	1	0

A total of 10 mice were examined per group.

AHN animals with hepatocellular necrosis; AHFC, animals with hepatocellular fatty change; AII, animals with inflammatory cell infiltration.

## Discussion

In this study, we confirmed the efficacy of SRF from the aerial part of *C. glandulosum* and examined its hepatoprotective effects using two animal models of hepatotoxicity.

CCl_4_ is a potent chemical hepatotoxin [20] that caused hepatocellular damage, as clearly indicated by the markedly elevated activities of serum enzymes (AST, ALT and TBIL) compared with non-treated control mice. Regarding the potential of SRF to prevent chemically-induced hepatotoxicity, the highest tested dose (0.20 g/kg body weight) was the most effective in this study. Although the ALT and AST activities were still higher with the highest dose of SRF than in the normal control animals, our histopathologic analyses revealed normal liver histology in group VI, and evidence of hepatotoxicity, such as hepatocellular necrosis, fatty changes, ballooning degeneration and infiltration of lymphocytes, was not detected.

Immune factors, such as autoimmune stimuli, virus infection or parasite infection, are the predominant causes of hepatic damage, especially under hepatitis [8]. The commonly used models of liver injury, which are often induced by chemicals, may not accurately reflect the clinical situation [21]. In this study, a BCG/LPS-induced liver injury model was used to investigate the hepatoprotective effects of SRF in mice. The elevated levels of ALT and AST were reduced after treatment with SRF, with the levels in group VI (0.20 g/kg body weight) reduced to the levels in the normal control group. Taken together, these findings suggest that the hepatoprotective effects of SRF may involve the ability for biomembrane protection against free radicals.

The induced liver damage was assessed by the TBIL activity in both models. Toxicity begins with changes to the endoplasmic reticulum, which result in the loss of metabolic enzymes located in the intracellular structures. SRF reduced the elevated TBIL levels, suggesting that SRF has the ability to stabilize biliary dysfunction in the mice liver during hepatic injury with CCl_4_ and BCG + LPS.

## Conclusion

SRF was hepatoprotective in animal models of chemical and immunological acute liver injury.

## Abbreviations

SRF: Sesquiterpene-rich fraction; CCl_4_: Carbon tetrachloride; CCl_3_: Trichloromethyl radical; BCG: Bacillus Calmette–Guerin; LPS: Lipopolysaccharide; AST: Aspartate aminotransferase; ALT: Alanine aminotransferase; TBIL: Total bilirubin; HE: Haematoxylin and eosin.

## Competing interests

The authors declare that they have no competing interests.

## Authors' contributions

HAA designed the study. WJY, MYH YQL, YM, LX and ZT performed the experiments. WJY, MYH, RPZ and XLX wrote the manuscript. All authors read and approved the final version of the manuscript.

## References

[B1] Day CP Alcohol and the liver Medicine 2007 35 22 25 10.1053/j.mpmed.2006.10.003

[B2] Abajo FJD Montero D Madurga M Rodriguez LAG Acute and clinically relevant drug-induced liver injury: a population based case–control study Br J Clin Pharmacol 2004 58 71 80 10.1111/j.1365-2125.2004.02133.x 15206996 PMC1884531

[B3] Joanne L Thanavaro ACNP-BC An overview of drug-induced liver injury J Nat Prod 2011 7 10 819 826

[B4] Gurpreet K Sarwar Alam M Zoobi J Kaleem J Mohammad A Evaluation of antioxidant activity of Cassia siamea flowers J Ethnopharmacol 2006 108 340 348 10.1016/j.jep.2006.05.021 16846707

[B5] Yang L Wang CZ Ye JZ Li HT Hepatoprotective effects of polyprenols from Ginkgo biloba L. leaves on CCl4-induced hepatotoxicity in rats Fitoterapia 2011 82 834 840 10.1016/j.fitote.2011.04.009 21596107

[B6] Mohamed E Shaker Zalata Khaled R Mehal Wajahat Z Shiha Gamal E Ibrahim Tarek M Comparison of imatinib, nilotinib and silymarin in the treatment of carbon tetrachloride-induced hepatic oxidative stress, injury and fibrosis Toxicol Appl Pharmacol 2011 252 165 175 10.1016/j.taap.2011.02.004 21316382 PMC3895503

[B7] Ferluga J Tuberculin hypersensitivity hepatitis in mice infected with Mycobacterium bovis (BCG) Am J Pathol 1981 105 82 90 7027805 PMC1903857

[B8] Zou YH Yang Y Li J Li WP Wu Q Prevention of hepatic injury by a traditional Chinese formulation, BJ-JN, in mice treated with Bacille-Calmette-GuÂ´erin and lipopolysaccharide J Ethnopharmacol 2006 107 442 448 10.1016/j.jep.2006.04.006 16697540

[B9] China Pharmacopoeia Committee Pharmacopoeia of the People’s Republic of China, the first division of 2010 edition 2010 Beijing: Chinese medicine and technology publishing house 291

[B10] Yl A Wu HK Aisa HA Kasimu RN Hang B Hepatoprotective activity evaluation of Cichorium glandulosum Nig J Nat Prod Med 2007 11 41 43

[B11] Wu HK Su Z Yang Y Isolation of three sesquiterpene lactones from the roots of Cichorium glandulosum Boiss. et Huet. by high-speed countercurrent chromatography J Chromatogr A 2007 1176 217 222 10.1016/j.chroma.2007.11.013 18037424

[B12] Wu HK Su Z YL A Isolation of esculetin from Cichorium glandulosum by high speed countercurrent chromatography Chem Nat Compd 2007 43 1 109 10.1007/s10600-007-0045-x

[B13] Yang WZ Wang H Shang J Feng F Xie N Chemical Constituents from Cichorium glandulosum Chin J Nat Med 2009 7 3 193 195 10.3724/SP.J.1009.2009.00193

[B14] Ren YL Zhou YW Chen XZ Ye YH Discovery, Structural Determination and Anticancer Activities of Lactucinlike Guaianolides Lett Drug Des Discov 2005 2 444 10.2174/1570180054771581

[B15] Sari N Dogan H Snyder JK US Patent 1997 5 663 196

[B16] Theodore AB Kelley CJ Karchesy Y Laurantos M Dinh PN Arefi AG Antimalarial activity of lactucin and lactucopicrin: sesquiterpene lactones isolated from Cichorium intybus L J Ethnopharmacol 2004 95 455 10.1016/j.jep.2004.06.031 15507374

[B17] Wesołowska Nikiforuk A Michalska K Kisiel W Chojnacka-Wójcik E Analgesic and sedative activities of lactucin and some lactucin-like guaianolides in mice J Ethnopharmacol 2006 107 254 10.1016/j.jep.2006.03.003 16621374

[B18] Christophe R Barbara S Nebojsa I Ilya R US Patent 2007 20 070 827 098

[B19] China Pharmacopoeia Committee Pharmacopoeia of the People’s Republic of China, the first division of 2010 edition 2010 Beijing: Chinese medicine and technology publishing house appendix VII A

[B20] Upur H Amat N Blažeković B Talip A Protective effect of Cichorium glandulosum root extract on carbon tetrachloride-induced and galactosamine-induced hepatotoxicity in mice Food Chem Toxicol 2009 47 2022 2030 10.1016/j.fct.2009.05.022 19477217

[B21] Shi YL Sun J He H Guo H Zhang S Hepatoprotective effects of Ganoderma lucidum peptides against D-galactosamine-induced liver injury in mice J Ethnopharmacol 2008 117 415 419 10.1016/j.jep.2008.02.023 18406549

